# Protecting blinded trials in electronic hospital systems

**DOI:** 10.1177/17407745211069985

**Published:** 2022-01-11

**Authors:** Matthew R Sydes, Wai Keong Wong, Ameet Bakhai, Nicola Joffe, Sharon B Love

**Affiliations:** 1 Institute of Clinical Trials and Methodology, MRC Clinical Trials Unit at UCL, London, UK; 2 British Heart Foundation Data Science Centre, London, UK; 3 Health Data Research UK, London, UK; 4 University College London Hospitals, London, UK; 5 Royal Free London NHS Foundation Trust, London, UK; 6 Amore Health Ltd, London, UK

Blinding (or masking) is considered pivotal for many clinical trials to prevent elements of bias and to prevent potential confounding. In a ‘blinded trial’, for a period of time, the intervention group allocation and/or other healthcare information is not disclosed to the participant, the investigator, the outcome assessor, other members of the site team, members of the central team, statisticians and/or combinations of these roles. Many blinded trials employ placebos or sham interventions which, in the case of some treatments, need to be produced or amended on site, or brought in. The decision to implement blinding should not be lightly taken as this has considerable practical and resource implications.^1–6^

Many healthcare settings now use electronic health record (EHR) systems, with government aspirations to be paper-light and improve healthcare green footprint.^7^ Access for patients to pertinent aspects of the EHR (‘patient portals’) is increasingly common in primary care settings and is developing in secondary care settings. This is good for patient autonomy, even if initially read-only.

Without careful consideration, information in the EHR may inadvertently allow elements of unblinding. For example, access to certain blood test results in the EHR could inadvertently reveal the treatment allocation to blinded staff or participants. This may be because of the treatment’s mode of action or a common side-effect (such as renal function change). In another setting, an early phase healthy volunteer study may give all participants the investigational treatment and blind them only to the allocated dose so that self-reporting of any side-effects is not informed by dose-related expectations. The dose needs to be visible to relevant staff but must be withheld from the participant in the EHRs. In addition, in a trial where the local pharmacy production unit makes up the active treatment while the placebo is imported, allowing all site staff to see in the EHR whether the production unit had been involved could effectively break the blind. Participants and relevant staff need to be protected from these results if they are to stay blinded.

Large, phase III trials, which might cost industry tens of millions of dollars, enrol multiple sites, internationally, in order for their treatment effects to be generalisable. Participating hospitals will use a variety of EHR systems, each with their own, local implementation and likely varying in their ability to restrict data access. Trials employing blinding crucially depend on outcome events being reported without bias. Rather than promoting high-quality research, EHRs designed poorly could delay, harm and, worse, invalidate research findings.

This is therefore an emerging and critical issue to tackle, especially for large-scale phase III or small-scale, rare disease trials where confounding could invalidate a treatment approach very early. These issues are raised by new technology predominantly because EHRs designers have deliberately focused on collation of data rather than on how it might need to be segregated. Healthcare settings must consider the needs of research when implementing a new EHR system and each EHR manufacturer needs to make it easy to enable appropriate new restrictions and controls to be applied by site teams.

In Table 1, we propose a series of simple actions that should be taken in general and for each trial at the implementation stage, prior to recruitment, and periodically thereafter. We anticipate that a ‘blinding matrix’ should be developed, including for implementation by local EHR team (see Supplemental Tables). The groups for the matrix should reflect the role groups available in the site’s EHR, the labelling of which may vary greatly across EHR systems and hospitals. Completing the matrix may require the central trial team to liaise with the local EHR team directly to ensure a correct mapping. If blinding cannot be well implemented, a site should not participate.

**Table 1.** Actions for trials employing blinding.

Trial coordination team (e.g. central clinical trials unit)
General: • Add text to protocol template’s section on site eligibility criteria (‘Site team willing and able to take steps to avoid unintentional unblinding through EHR system’) • Add text to protocol template’s section on quality assurance and quality control (QA/QC) about need for trial teams (perhaps, monitors) to check EHR process early in trial and periodically thereafter
Specific trials: • Trial Development Group to discuss with approach groups whether blinding is particularly needed • Develop clear list of who in a blinded/masked trial needs to be blinded and to what they must be blinded (Supplemental Tables 1 and 2) • Add item on Risk Assessment (or Risk Register) for blinded trials to flag need to work with sites EHR teams to take steps to avoid unintentional unblinding • Make clear that implementation of blinding at site (including in EHR) has been delegated from Sponsor in protocol and Site Agreement • Add item on Site Initiation Checklist re-training site staff on interacting with EHR, particular with need to label, in the EHR, relevant clinical encounters as relating to a trial • Add item to Site Initiation Checklist re-ensuring site has relevant processes to put in place • Discussion at Site Initiation visit about how EHR is set up and what relevant people will have access to. Do not approve site until discussion finalised and any actions agreed • Add checkpoint on Monitoring Plan re-ensuring that site staff are trained to appropriately label clinical encounters in the EHR as relating to a trial • Add checkpoint on Monitoring Plan to assess that blinding is being adhered to identify which SOP or WI to follow if blinding is inadvertently broken
Site implementation team (e.g. hospital)
Specific trials: • Site Principal Investigator (PI) to identify who takes ownership of checking and implementing the local blinding matrix • Site PI to raise with EHR team that a blinded trial is being set up. Note that many site staff team members will be involved in set-up before the EHR team is usually involved • Implement site’s standard SOP for dealing with blinded trials, ensuring a compliant workflow • Site PI or delegate to ensure that blinding matrix is reviewed by all relevant parties and that blinding can be reasonably protected • If drug trial, ensure that pharmacy has processes in place to ensure information is withheld from relevant staff members (e.g. blinded and unblinded pharmacist model), particularly if production unit/development/packaging is at site • If non-drug trial, ensure that relevant department has processes in place • Site PI and site EHR team to train other site staff to label, in the EHR, clinical encounters as relating to a trial or trial-relevant in any way • Site EHR to flag on system each patient that is on the trial, that the trial is blinded and whether blinding remains in place for individual participant (e.g. allocation revealed through emergency unblinding procedure) • Site PI and site EHR team to check early in trial that blinding is not obviously being broken for key role functions or through relevant reports. Key documents to check include discharge summaries and GP letters • Site PI to consider practical implementation of blinding with relevant shift teams (e.g. night teams) • Site EHR team to develop scripts to test whether the blinding can be protected in blinded trials. These may be bespoke to trial (following the needs of Supplemental Table 1) or made generic across trials fitting some blinding matrix • If a piece of information needs to be blinded to every site member in the matrix, consider not putting it in the EHR

EHR: electronic health record; GP: general practitioner; PI: (site) principal investigator; SOP: standard operating procedure; WI: working instruction.

Maintaining blinding of treatment allocation can also be challenged in traditional, paper records. Although central and site teams should be more well-versed in addressing these, our tables may still prove helpful.

Groups and roles in EHR systems are complex and varied. These simple steps, employed for every blinded clinical trial, will help to protect clinical trials from the potential for inadvertent unblinding.

## Supplemental Material

sj-pdf-1-ctj-10.1177_17407745211069985 – Supplemental material for Protecting blinded trials in electronic hospital systems Supplemental material, sj-pdf-1-ctj-10.1177_17407745211069985 for Protecting blinded trials in electronic hospital systems by Matthew R Sydes, Wai Keong Wong, Ameet Bakhai, Nicola Joffe and Sharon B Love in Clinical Trials
