# Supplementary material for: Protecting blinded trials in electronic hospital systems
Source: Clin Trials. 2022 Jan 11;19(2):231–3. doi: 10.1177/17407745211069985 (PMC9036147; doi:10.1177/17407745211069985)
Supplement: sj-pdf-1-ctj-10.1177_17407745211069985 – Supplemental material for Protecting blinded trials in electronic hospital systems [file sj-pdf-1-ctj-10.1177_17407745211069985.pdf]

Supplement Table 1: Template trial-level blinding matrix for site (with example rows)
-- For implement across site including in EHR and patient access apps

Information to blind	Until when (if blinded)	Participants	Monitor (visiting site)	Site PI & Site investigators	Nursing team	Treatment administrator	Pharmacist	Site diagnostics team	Site EHR team	Other role [Add]	GP	[Other role: Add]
	Group	1	2	2	2	2	2	2	2	2	3	4
Allocation to intervention group	End of trial											
Allocated treatment dose	End of trial											
Blood test results (specify)	End of trial											
Scan results	End of trial											
Discharge summary	End of trial											
GP letter	End of trial											
[Other information: add]	[Add]											

Key

Column groups
1 = Participants
2 = Site Implementation Team
3 = Potential Recipients of Information

Cells
B = Blinded
U = Unblinded

Use
Delegated team members (including EHR team members) should start with Participants when implement the blinding strategy. If blinding cannot be achieved for Participants, do not proceed with trial as-is; else assess for Site Implementation Team. If blinded can be achieved for Site Implementation Team, do not proceed with trial as-is; else assess for Potential Recipients of Information, etc

Supplement Table 2: Template trial-level blinding matrix for trials unit (with example rows)
-- For implement across site including in EHR and patient access apps

	Group	A	A	A	A	A	A
**Information to blind**	**Until when (if blinded)**	**Trials unit ops team**	**Blinded statistician**	**Unblinded statistician**	**Clinical reviewers**	**Monitor (at office)**	**[Other role: Add]**
Allocation to intervention group	End of trial						
Allocated treatment dose	End of trial						
Blood test results (specify)	End of trial						
Scan results	End of trial						
Discharge summary	End of trial						
GP letter	End of trial						
[Other information: add]	[Add]						

Key

Column groups
A = Trials unit staff

Cells
B = Blinded
U = Unblinded

Note
Unblinded statistician is one that runs interim analyses and can see accumulating, comparative data by allocated group; Blinded statistician does not see accumulating, comparative data
